# Complete Genome Sequence of *Klebsiella pneumoniae* Phages SopranoGao, MezzoGao, and AltoGao

**DOI:** 10.1128/genomeA.01009-17

**Published:** 2017-11-09

**Authors:** Sarah Gao, Sara B. Linden, Daniel C. Nelson

**Affiliations:** a Montgomery Blair High School, Silver Spring, Maryland, USA; b Institute for Bioscience and Biotechnology Research, University of Maryland, Rockville, Maryland, USA

## Abstract

The *Klebsiella pneumoniae* phages SopranoGao, MezzoGao, and AltoGao were isolated from the Seneca Wastewater Treatment Plant in Germantown, MD. The following reports the complete genome sequence of these bacteriophages and describes their major features.

## GENOME ANNOUNCEMENT

*Klebsiella pneumoniae* is a Gram-negative bacterium in the *Enterobacteriaceae* family and is increasingly associated with nosocomial infections in humans (1). Here, we describe the complete genomes of SopranoGao, MezzoGao, and AltoGao bacteriophages active against *K. pneumoniae*. All three phages were isolated from the Seneca Wastewater Treatment Plant in Germantown, MD, USA (39°14′27.6″N, 77°27′57.4″W), and grown on *K. pneumoniae* ATCC 51503 as the host strain. Genomic DNA was isolated from each phage and digested with restriction enzymes (EcoRI, BamHI, HindIII), confirming three distinct phages. Phage DNA was sequenced at the Genomic Sciences Laboratory, North Carolina State University, on an Illumina next-generation platform. Quality-controlled, trimmed reads were assembled into single contigs from 689-fold (SopranoGao), 1,587-fold (MezzoGao), and 1,552-fold (AltoGao) coverage of the genomes using GS De Novo Assembler (Newbler) version 2.7 and Consed version 29.0 (2). SopranoGao, the largest genome, contained 61,646 bases, MezzoGao contained 49,809 bases, and AltoGao contained 43,012 bases. AltoGao had defined ends with terminal repeats, whereas SopranoGao and MezzoGao did not contain ends. Therefore, regions upstream of the terminases were chosen as the canonical start sites for these genomes. All genes were predicted using Glimmer and GeneMarkS and corrected using DNA Master (http://phagesdb.org/DNAMaster/).

Analysis of the SopranoGao, MezzoGao, and AltoGao genomes found 77, 77, and 53 open reading frames, respectively, and G+C contents of 57.26%, 50.97%, and 53.29%, respectively. The start codon frequencies were similar for ATG (86%, 93%, and 87%), GTG (13%, 4%, and 6%), and TTG (1%, 3%, and 7%) between SopranoGao, MezzoGao, and AltoGao, respectively. No tRNA genes were detected in any of the genomes by the tRNAscan-SE program (3). The putative protein function of the open reading frames was determined by protein BLAST (4). The majority of the predicted genes contained hypothetical proteins, although well-conserved phage proteins were found in all genomes. For example, all genomes contained genes for holins, endolysins, tail fiber proteins, terminases/maturases, and major capsid proteins. DNA primases and helicases were found in MezzoGao and AltoGao, as were spanins. In contrast, SopranoGao contained a lytic protein (orf2) in addition to the endolysin (orf5), but it is not clear if this gene represents a spanin or a second endolysin.

By nucleotide BLAST search of the genomes, the nearest neighbor (identity 90%) of SopranoGao is *Erwinia* phage PEp14, which is classified as belonging to the *Podoviridae* family. The nearest neighbors (identity range 96 to 98%) of MezzoGao are *Klebsiella* phage Sushi, *Klebsiella* phage KP36, and *Klebsiella* phage 1513. All of these phages are classified as belonging to the *Siphoviridae* family, *Tunavirinae* subfamily, and *Kp36virus* genus. The nearest neighbors (identity range 88 to 90%) of AltoGao are *Klebsiella* phage vB_KpnP_SU503, *Klebsiella* phage KP34, *Klebsiella* phage vB_KpnP_KpV41, and *Klebsiella* phage vB_KpnP_SU552A. All of these phages are classified as belonging to the *Podoviridae* family and *Autographivirinae* subfamily. Thus, MezzoGao and AltoGao are members of groups of closely related *K. pneumoniae* phages.

### Accession number(s).

The complete and annotated genomes of bacteriophages SopranoGao, MezzoGao, and AltoGao have been deposited in GenBank under the accession numbers MF612073, MF612072, and MF612071, respectively.
